# RHOV promotes lung adenocarcinoma cell growth and metastasis through JNK/c-Jun pathway

**DOI:** 10.7150/ijbs.59939

**Published:** 2021-06-22

**Authors:** Deyu Zhang, Qiwei Jiang, Xiangwei Ge, Yanzhu Shi, Tianxing Ye, Yue Mi, Tian Xie, Qihong Li, Qinong Ye

**Affiliations:** 1 Department of Medical Molecular Biology, Beijing Institute of Biotechnology, Beijing 100850, P.R. China.; 2 Department of Oncology, Chinese PLA General Hospital, Beijing 100853, P.R. China.; 3 Medical College, Guizhou University, Guiyang 550025, P.R. China.; 4 College of Medicine, Yanbian University, Yanji 133000, P.R. China.; 5 Department of Stomatology, The Fifth Medical Centre, Chinese PLA General Hospital, Beijing 100071, P.R. China.

**Keywords:** Lung adenocarcinoma, metastasis, JNK/c-Jun pathway, RHOV, bioinformatics

## Abstract

Lung adenocarcinoma (LUAD) is a common type of lung cancer with high frequent metastasis and a high death rate. However, genes responsible for LUAD metastasis are still largely unknown. Here, we identify an important role of ras homolog family member V (RHOV) in LUAD metastasis using a combination of bioinformatic analysis and functional experiments. Bioinformatic analysis shows five hub LUAD metastasis driver genes (RHOV, ZIC5, CYP4B1, GPR18 and TCP10L2), among which RHOV is the most significant gene associated with LUAD metastasis. High RHOV expression predicted shorter overall survival in LUAD patients. RHOV overexpression promotes proliferation, migration, and invasion of LUAD cells*,* whereas RHOV knockdown inhibits these biological behaviors. Moreover, knockdown of RHOV suppresses LUAD tumor growth and metastasis in nude mice. Mechanistically, RHOV activates Jun N-terminal Kinase (JNK)/c-Jun signalling pathway, an important pathway in lung cancer development and progression, and regulates the expression of markers of epithelial-to-mesenchymal transition, a process involved in cancer cell migration, invasion and metastasis. RHOV-induced malignant biological behaviors are inhibited by pyrazolanthrone, a JNK inhibitor. Our findings indicate a critical role of RHOV in LUAD metastasis and may provide a biomarker for prognostic prediction and a target for LUAD therapy.

## Introduction

Lung carcinoma is one of malignant cancers with the highest incidence and the worst prognosis in worldwide 1. Most lung carcinoma patients are diagnosed at advanced stage with metastatic disease 2, 3. Among all lung carcinoma subtypes, lung adenocarcinoma (LUAD) is the most common and aggressive subgroup 2. The pathophysiological mechanisms of high metastasis rate of LUAD remain poorly understood. Several factors, such as immune escape 4, genetic mutation 5, and pathological and molecular alterations 6, were reported to be associated with metastasis of LUAD. Considering the inadequate understanding of LUAD metastasis, we need to explore novel targets.

Ras Homolog (Rho) GTPases belong to the Ras superfamily of small GTPases, and play important roles in regulation of cell cycle, cell adhesion and migration 7. Ras homolog family member V (RHOV) is considered an atypical member of Rho GTPases because of unique N-terminal and C-terminal regions. RHOV was reported to promote neural crest development during vertebrate embryogenesis 8. In zebrafish, RHOV has ability to activate (p21-activated kinases) PAK1, thus promoting early vertebrate development 9. Although RHOV expression was shown to be upregulated in lung carcinoma 10, the biological function of RHOV in cancer is unclear.

In this study, we demonstrate that RHOV may be associated with metastasis in LUAD through bioinformatics. We show that silencing of RHOV reduces LUAD cell proliferation, migration, invasion, and metastasis *in vitro* and *in vivo*. Mechanistically, RHOV activates Jun N-terminal Kinase (JNK)/c-Jun signalling pathway, a critical pathway in lung cancer development and progression, and modulates the expression of markers of epithelial-to-mesenchymal transition (EMT), a process involved in cancer cell migration, invasion and metastasis.

## Materials and Methods

### Plasmids, cell lines and reagents

The FLAG-tagged RHOV eukaryotic expression vector was constructed using the PCR-amplified RHOV fragment and pcDNA3.0 (Invitrogen) with FLAG tag. The lung cancer cell lines A549 and H1299 were purchased from the American Type Culture Collection. The target sequence of RHOV shRNA was 5'-GGCTATTCTCAGTGCCATT-3'. Lentivirus was produced using pSIH-H1-Puro (System Biosciences) carrying RHOV shRNA according to the manufacturer's instructions, and was infected into A549 and H1299 lung cancer cells to generate individual clones. Anti-JNK, anti-phospho-JNK (pJNK), anti-c-Jun, anti-phospho-c-Jun and anti-RHOV were purchased from Proteintech; Anti-E-cadherin, anti-N-cadherin, anti-Snail and anti-Slug were purchased from BD Biosciences.

### Data collection

The data of 535 LUAD tissue samples and 59 adjacent tissue samples were obtained from The Cancer Genome Atlas (TCGA-LUAD, www.cancergenome.nih.gov) 11. The AJCC tumor-node-metastasis (TNM) staging system was utilized in definition of metastatic predisposition. Two hundred thirty one LUAD tissues with N0 and M0 stage were identified as non-metastatic predisposition, and 185 LUAD tissues with N1-3 or M1 stage were identified as metastatic predisposition.

### Identification of metastasis driver genes (MDGs) and construction of Protein-protein interaction (PPI) network

To investigate differentially expressed genes (DEGs) between LUAD tissues with non-metastatic predisposition and LUAD tissues with metastatic predisposition in TCGA-LUAD dataset, we used the limma R package 12. DEGs were set as follows: False Discovery Rate (FDR) <0.05 and |log_2_ fold change (FC)|>1. Among DEGs, a PPI network of MDGs was built by the Search Tool for the Retrieval of Interacting Genes (STRING, www.string-db.org/), and was organized and visualized by Cytoscape software.

### Construction and evaluation of LUAD-specific prognostic model

Univariable Cox regression analysis was performed to explore the relationships between MDGs and overall survival (OS) of 505 LUAD samples from TCGA-LUAD dataset. Multivariate Cox regression analysis was performed to establish a prognostic signature for each LUAD sample. The risk score was calculated as follows: Risk score = ∑βi×expRNAi 13. The cases were ranked into low- and high-risk groups based on the median score, and the survival curves were plotted. Receiver operating characteristic (ROC) curve analysis was utilized to assess the prognostic signature.

### Analysis of Prognostic Value

Gene Expression Profiling Interactive Analysis (GEPIA, http://gepia.cancer-pku.cn/) was used for analysis of the associations between MDGs and overall survival (OS) or disease-free survival (DFS) of LUAD patients from TCGA-LUAD dataset.

### Cell culture and Transfection

A549 and H1299 cells were routinely cultured in Ham's F12 Medium or RPMI-1640 Medium containing 10% fetal bovine serum (FBS) at 37 °C in a humidified atmosphere with 5% CO2. For plasmid transfection, cells were inoculated into 6-well plates at a cell density of about 70-80% and transfected after adherence to the wall. The reagents for transfection of plasmids were Lipofectamine 3000 (Promega) and were used according to the manufacturer's instructions. Cell culture medium was replaced after 4-6 h, and the transfected cells were collected after 24-48 h.

### Cell proliferation and colony formation assays

Cell proliferation assays were measured using the CCK-8 Kit (Dojindo Laboratories, Japan) according to the manufacturer's instructions. Cells were seeded in 96-well plates at a density of 3×10^3^/well, and 3 parallel wells were set in each group. DMEM containing 10% CCK8 reagent was added to each well at the same time point every day, and cultured at 37 °C for 1 h. The OD450nm values were measured using a microplate reader. For colony formation assays, cells were inoculated in 6-well plate with 3×10^3^ cells. After about two weeks, the colonies were treated with 4% paraformaldehyde and 0.5% crystal stained purple was kept for 30 minutes. Colonies larger than 1.5 mm in diameter were counted.

### Wound-healing and transwell assays

Cells were inoculated in 6-well plates for cell transfection. After the cell density reached 80-90%, the pipette head was used for scratching. The floating cells were washed with PBS buffer. Cultured cells were grown for 16 h to allow wound closure. The wound healing rates were measured and compared to the width at 0 h. Transwell assay was used to detect cell invasion ability. Cells were collected and suspended in serum-free medium. Six hundred microliters of medium containing 20% FBS was added to the 24-well plate, and about 200 uL cell suspension was added to the upper chamber with matrigel, and cultured for 16 h in an incubator at 37℃. The chamber was fixed in 4% paraformaldehyde and then stained in 0.1% crystal violet solution for 30 minutes, respectively. A cotton swab was used to wipe the cells and matrigel out of the chamber. The images were observed under a microscope, and the number of invasive cells was calculated using the Image J software.

### Tumor growth and metastasis in nude mice

Animal experiments were performed with protocols approved by the Institutional Animal Care Committee at Beijing Institute of Biotechnology. Ten million A549 cells stably expressing RHOV shRNA or control shRNA were injected subcutaneously in the right flank of each nude mice. Tumor size was examined at the indicated times. The mice were euthanized at the indicated time. Excised tumors were perserved in liquid nitrogen.

For tumor metastasis analysis, 1 × 10^7^ A549 cells stably expressing RHOV shRNA or control shRNA were injected into the tail vein of each nude mice. After euthanasia at the indicated time, all lung metastatic foci were investigated histologically.

### Statistical analysis

SPSS 19.0 and GraphPad Prism 8.0 were used for statistical analysis. Student's *t* test was applied to data comparison between two groups. Analysis of variance (ANOVA) was used for comparison among several groups. Student's *t* test, Chi-square test and Mann-Whitney Test was adopted to analyze the relationship between clinicopathological characteristics and metastatic predisposition in LUAD patients. All experiments were independently repeated for 3 times, and the data were expressed as mean ± SD. *P* < 0.05 was the threshold of statistical significance.

## Results

### Identification of RHOV as a critical gene associated with LUAD metastasis

**Figure 1:**
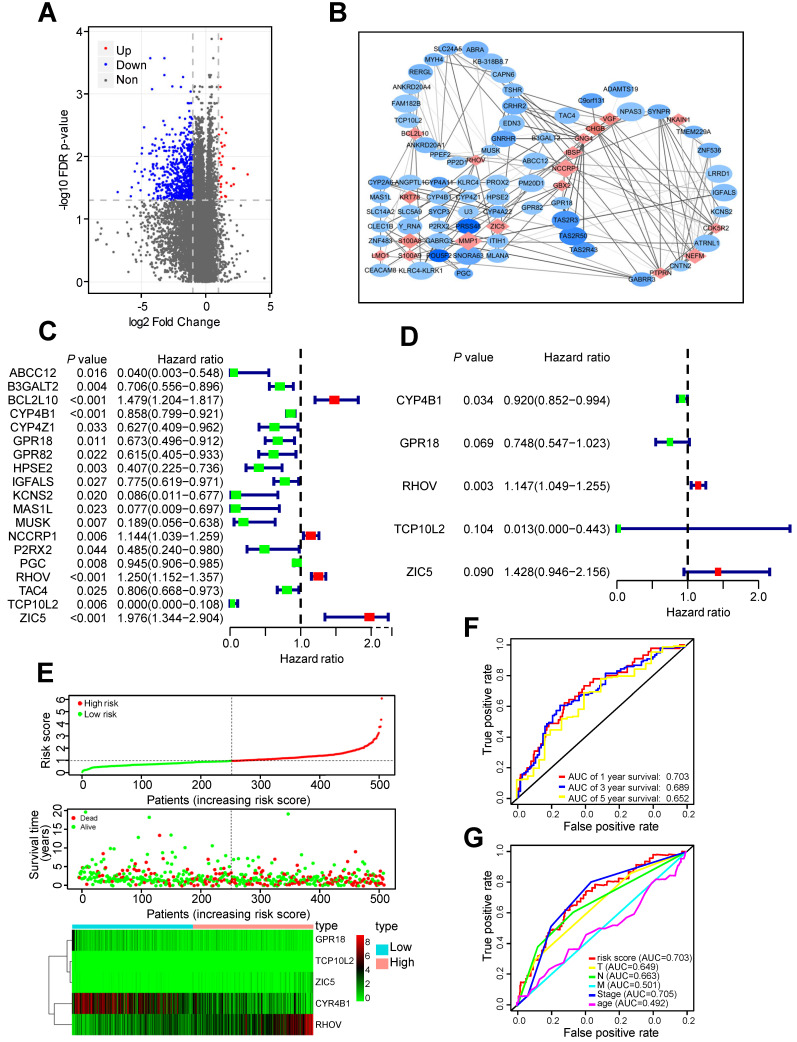
** Identification of RHOV as the most significant gene associated with LUAD metastasis. (A)** The volcano plot showing differentially expressed genes between tumor tissues with non-metastatic predisposition and tumor tissues with metastatic predisposition from TCGA-LUAD dataset. **(B)** A PPI network analysis of 83 MDGs. **(C and D)** The hazard ratio (HR) and 95% confidence interval of genes were calculated by univariate (C) and multivariate (D) Cox regression analysis. **(E)** The distribution of risk score, survival status and gene expression of LUAD patients from TCGA-LUAD dataset. **(F and G)** ROC analysis of the prognostic signature with 1-, 3- and 5-year overall survival and clinicopathological features with 1-year overall survival of LUAD patients.

Two hundred thirty one LUAD tissues with non-metastatic predisposition and 185 LUAD tissues with metastatic predisposition from the TCGA-LUAD dataset were analyzed. The main clinical characteristics were presented in Table S1. Compared to non-metastasis group, 30 genes were upregulated and 665 genes were downregulated in metastasis group (Figure 1A). In order to screen out the most important and largest functional module, these differentially expressed genes (DEGs) were further selected as metastasis driver genes (MDGs) by the STRING online database, and a PPI network for 83 MDGs was built (Figure 1B).

These 83 MDGs were chosen for further analysis of the association between MDGs and patients' outcomes. Univariable Cox regression analysis was utilized to identified 19 MDGs with significant prognostic values (Figure 1C). Subsequently, multivariate Cox regression analysis was utilized to screen out five hub MDGs: RHOV, Zic family member 5 (ZIC5), cytochrome P450 family 4 subfamily B member 1 (CYP4B1), G protein-coupled receptor 18 (GPR18), and t-complex 10 like 2 (TCP10L2). A five-mRNA based prognostic signature was constructed and the prognostic risk score for each LUAD patient was as follows: (0.137*RHOV + 0.356* ZIC5 - 0.083* CYP4B1 - 0.29* GPR18 - 4.37* TCP10L2). The concordance index of this formula was 0.68, indicating a certain predictive effect. Among these five MDGs, RHOV was the most significant independent prognostic factor (*P* value = 0.003, Hazard ratio = 1.147), and may play an essential role in metastasis and prognosis of LUAD patients (Figure 1D).

Moreover, we divided 504 LUAD patients into low- and high-risk groups based on the median risk score. Survival analysis and the expression of five MDGs in different subgroups was performed (Figure 1E). Patients in high-risk groups had a shorter OS time and higher death rate than those in low-risk groups. In addition, the area under the ROC curve (AUC) values of the prognostic signature for 1-, 3-, and 5-year survival rates were 0.703, 0.689, and 0.652, respectively (Figure 1F). The AUC analysis revealed that prognostic signature was a substitute for other clinical factors in predicting LUAD patients' prognosis (Figure 1G).

### The validation of RHOV and other four hub MDGs in TCGA-LUAD dataset

**Figure 2:**
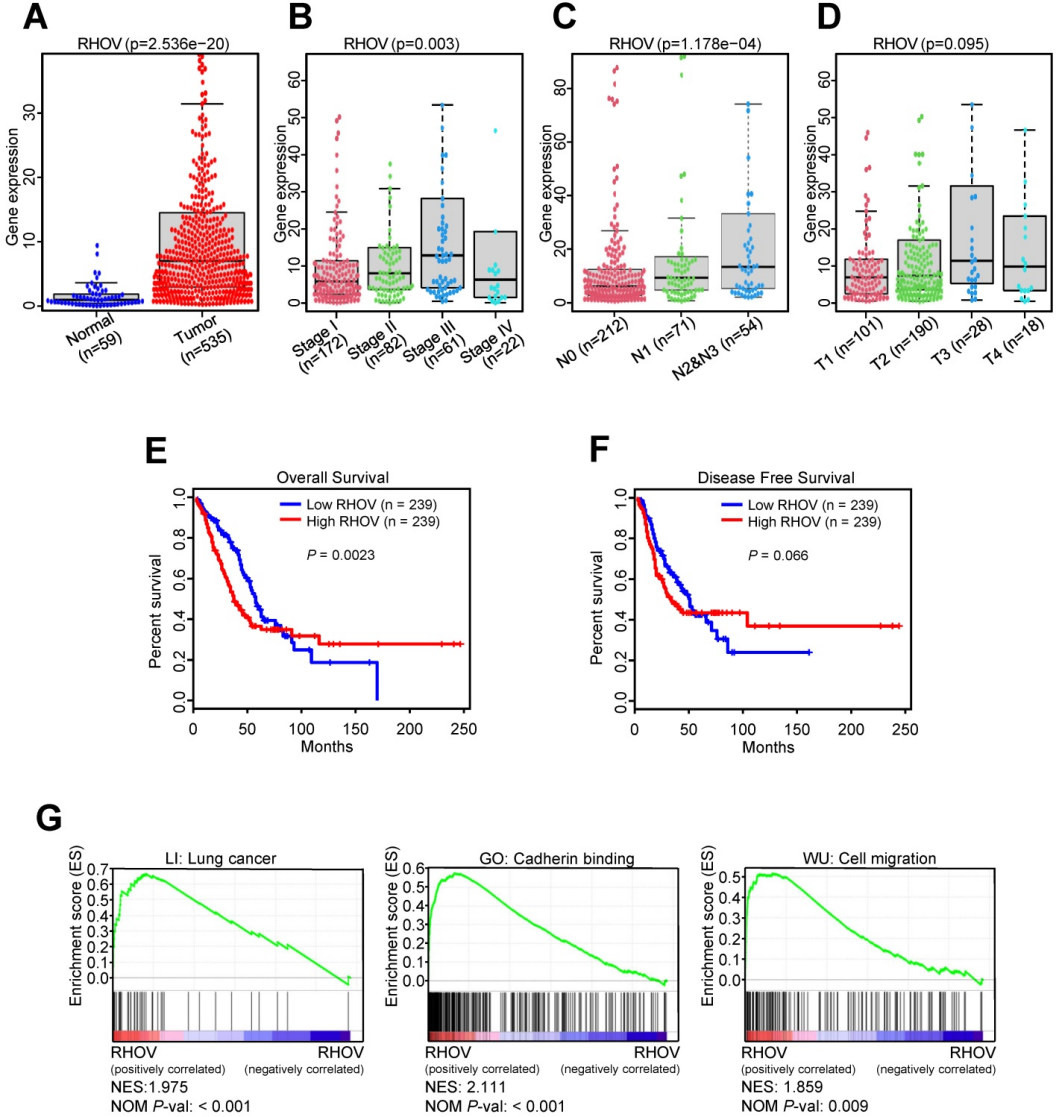
** The validation and function prediction of RHOV in LUAD patients from TCGA-LUAD dataset. (A-D)** The associations between RHOV mRNA expression and clinicopathological features in LUAD patients from TCGA-LUAD dataset. **(E and F)** The associations between RHOV mRNA expression and overall survival (E) and disease-free survival (F) in LUAD patients from TCGA-LUAD dataset. **(G)** GSEA was conducted to predict the function of RHOV in LUAD.

Next, we investigated the associations between RHOV and clinicopathological features of LUAD patients from TCGA-LUAD dataset. Compared to normal tissues, the mRNA expression level of RHOV was significantly increased in LUAD tissues (Figure 2A). RHOV mRNA expression was also increased in those with advanced clinical stage (Figure 2B), lymph node metastasis (Figure 2C), and large tumor size (Figure 2D). However, due to the small sample size, the association between RHOV and distant metastasis could not be analyzed. The associations between other four hub MDGs and clinicopathological features were also examined. Compared to normal tissues, CYP4B1 mRNA level was significantly decreased (Figure S1A), and GPR18, TCP10L2 and ZIC5 mRNA level were significantly increased in LUAD tissues (Figure S1B-D). CYP4B1 mRNA level was decreased in those with advanced clinical stage (Figure S1E). There were limited clinical values of GPR18, TCP10L2, and ZIC5 mRNA level, because of non-consistent expression level or non-significance in different clinical stages (Figure S1F-H).

In the first 50 months of LUAD, high RHOV mRNA expression was associated with shorter OS in LUAD patients (Figure 2E). After 50 months, the negative correlation between RHOV mRNA expression and OS disappeared in LUAD patients. These results suggest that RHOV may play a key role as an oncogene in the early LUAD. As for the relation between RHOV and DFS in LUAD, similar phenomenon was found (Figure 2F). The associations between other four hub MDGs and prognosis of LUAD patients were also analyzed. CYP4B1 mRNA expression was positively correlated with OS in LUAD patients, and GPR18 and ZIC5 mRNA expression were negatively correlated with OS (Figure S2A-C). There was no significant correlation between mRNA expression of these MDGs and DFS in LUAD patients (Figure S2D-F).

To explore the function of RHOV in LUAD, gene set enrichment analysis (GSEA) was performed. The result showed that several pathways associated with LUAD metastasis, such as cadherin binding and cell migration, were significantly enriched (Figure 2G).

### RHOV promotes LUAD cell proliferation, migration and invasion *in vitro*

**Figure 3:**
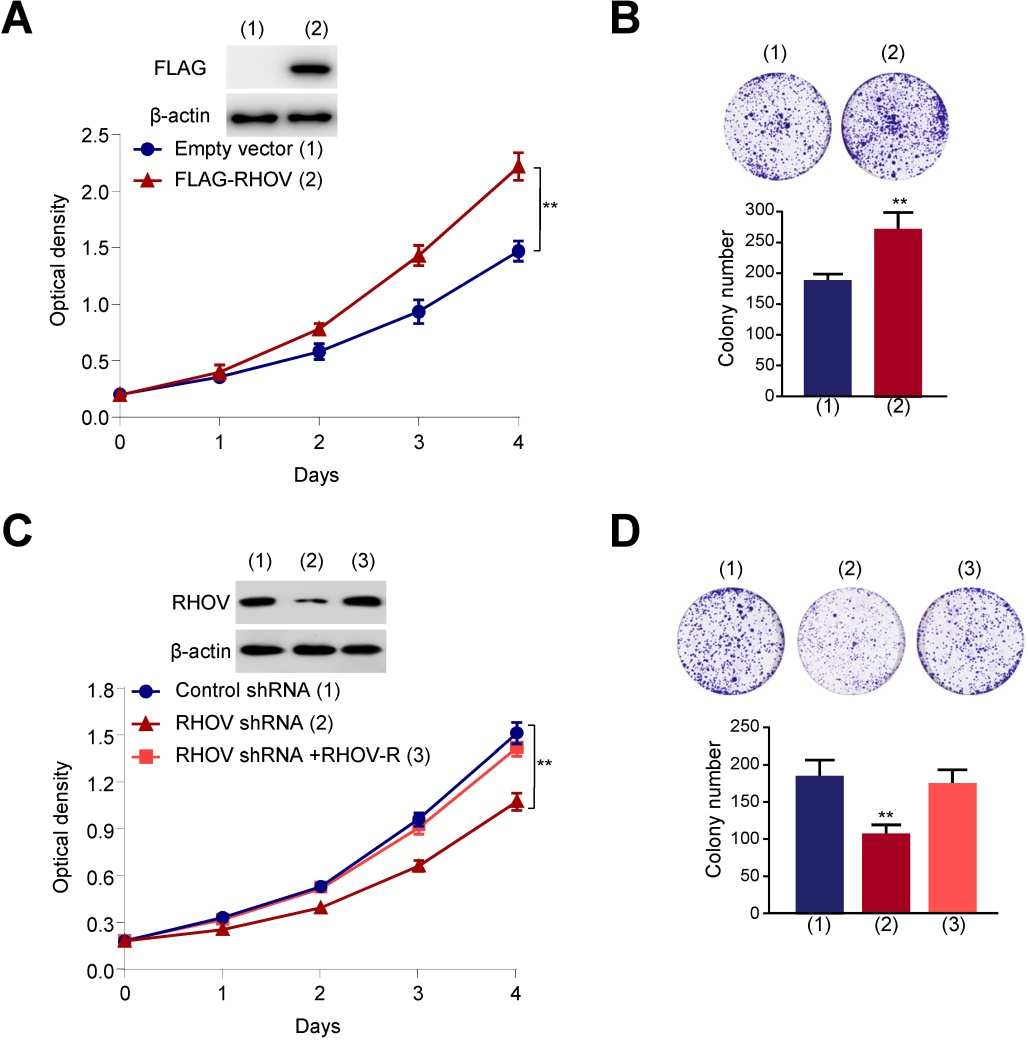
** RHOV promotes cell proliferation in A549 cells. (A)** A549 cells infected with FLAG-tagged RHOV plasmid or empty vector were grown in regular medium. Cell numbers were detected at the indicated times. Representative immunoblot demonstrates the expression of FLAG-RHOV. **(B)** Colony formation assays for A549 cells infected as in (A). **(C)** A549 cells were infected with control shRNA, RHOV shRNA or RHOV shRNA plus siRNA-resistant RHOV (RHOV-R), and analyzed as in (A). Representative immunoblot shows RHOV expression. **(D)** Colony formation assays for A549 cells infected as in (C). Data shown are mean ± SD of triplicate measurements with similar results (*P < 0.05, **P < 0.01).

Next, we investigated the function of RHOV on proliferation, migration and invasive capacity of LUAD cells. We chose two LUAD cell lines (A549 and H1299 cells) to test the function of RHOV. The expression level of RHOV in H1299 cells was only a little higher than that in A549 cells (Figure S3A). Thus, we used the same cell line to overexpression or knockdown of RHOV. Cell proliferation and colony formation assays revealed that FLAG-tagged RHOV-overexpressing A549 cells grew faster than empty vector-containing cells (Figure 3A and 3B). In contrast, RHOV knockdown reduced proliferation and colony formation of A549 cells, and reexpression of RHOV in the RHOV knockdown cells rescued this effect (Figure 3C and 3D). Similar effects were observed in H1299 cells infected with FLAG-RHOV or RHOV shRNA plasmids (Figure S3B-E).

**Figure 4:**
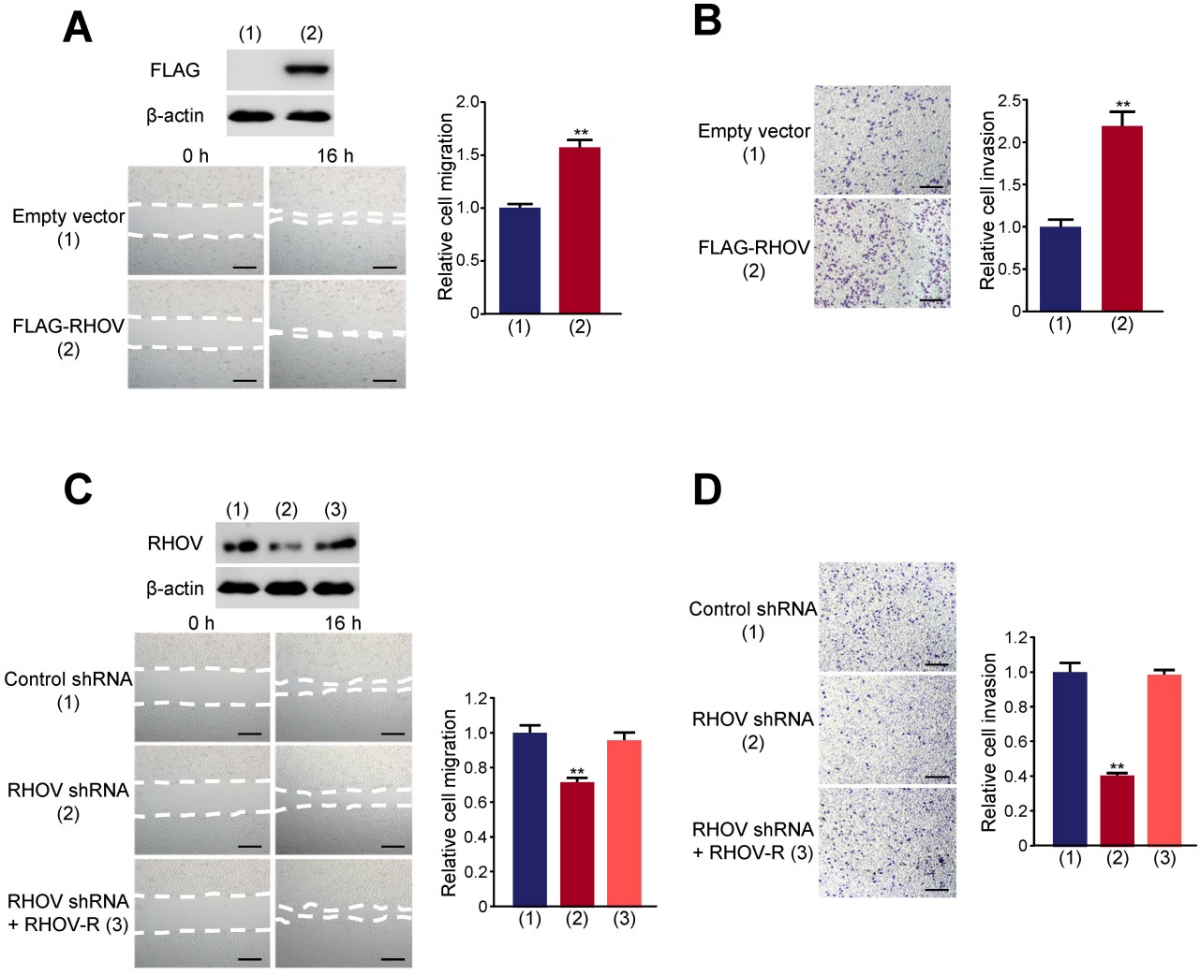
** RHOV promotes migration and invasion in A549 cells. (A and B)** Migration (A) and invasion (B) of A549 cells overexpressing FLAG-tagged RHOV plasmid or empty vector were evaluated by wound-healing assays and transwell assays, respectively. Scale bar: 100 µm. Representative immunoblot demonstrates the expression of FLAG-RHOV. **(C and D)** Migration (C) and invasion (D) of A549 cells infected with control shRNA, RHOV shRNA or RHOV shRNA plus siRNA-resistant RHOV (RHOV-R) were evaluated by wound-healing assays and transwell assays, respectively. Scale bar: 100 µm. Representative immunoblot shows RHOV expression. Data shown are mean ± SD of triplicate measurements with similar results (*P < 0.05, **P < 0.01).

Wound-healing showed that RHOV overexpression promotes A549 cell migration (Figure 4A). Transwell assays demonstrated that RHOV overexpression increased the number of invaded A549 cells (Figure 4B). In contrast, RHOV knockdown reduced A549 cell migration and invasion, and reexpression of RHOV in the RHOV knockdown cells rescued this effect (Figure 4C and 4D). Similar effects were observed in H1299 cells (Figure S4). These data demonstrated that RHOV promotes LUAD cell proliferation, migration and invasion *in vitro*.

### JNK pathway is responsible for RHOV regulating LUAD cell proliferation, migration, invasion as well as the expression of EMT-related genes

**Figure 5:**
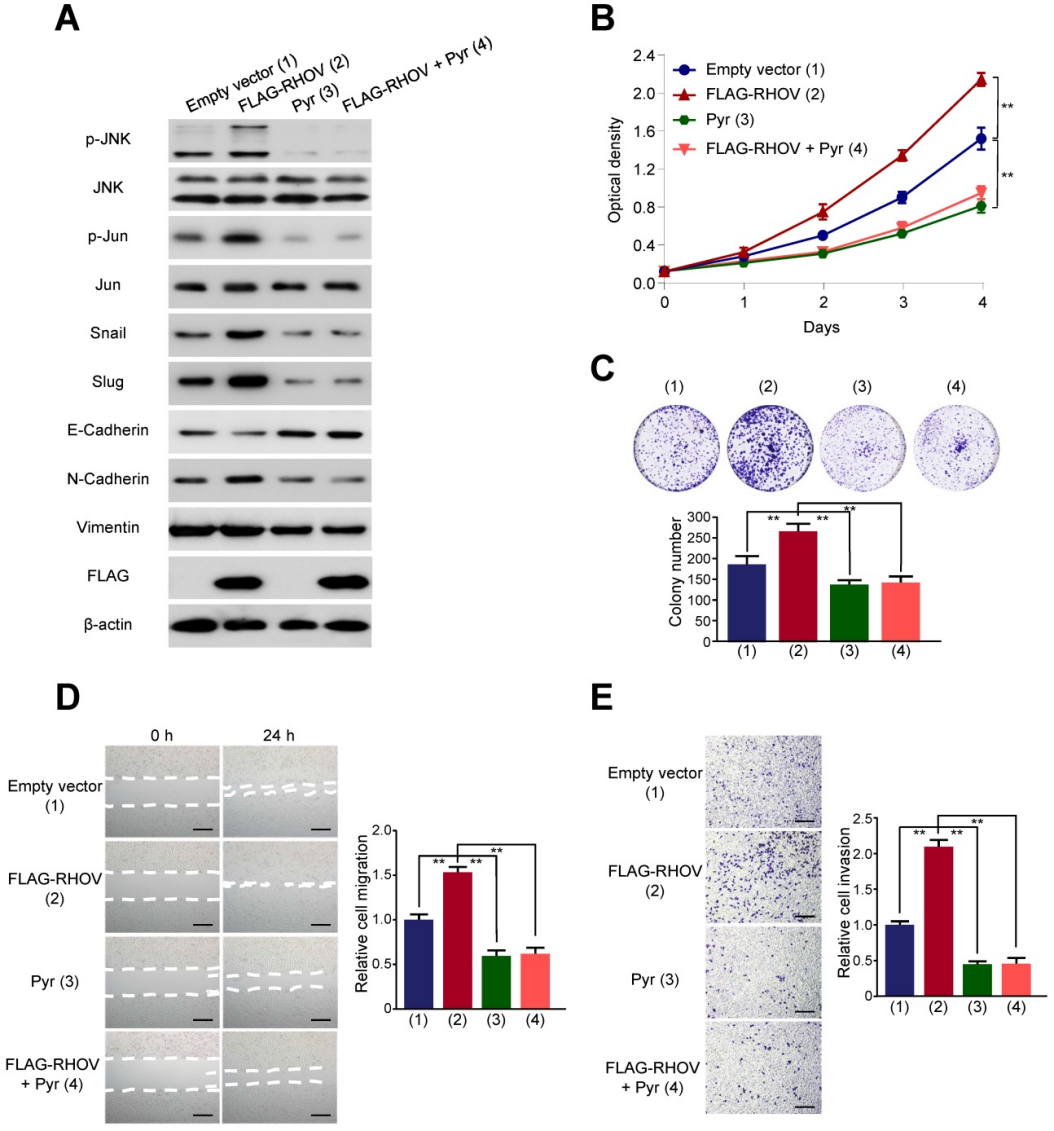
** RHOV regulates expression of EMT-related proteins via JNK pathway. (A)** Western blot analysis of A549 cells infected with FLAG-tagged RHOV plasmid and treated with the JNK inhibitor pyrazolanthrone (10 µM). **(B and C)** A549 cells were treated as in (A), and cell numbers (B) and cell colonies (C) were detected at the indicated times. **(D and E)** Migration (D) and invasion (E) of A549 cells treated as in (A) were evaluated by wound-healing and transwell assays, respectively. Scale bar: 100 µm. Data shown are mean ± SD of triplicate measurements with similar results (*P < 0.05, **P < 0.01).

It has been reported that RHOV activate JNK pathway in human embryonic kidney 293 cells and rat pheochromocytoma PC12 cells 14, 15. However, whether RHOV stimulates JNK pathway in human cancer cells is unknown. JNK pathway was known to be an essential role in lung cancer cell survival and metastasis 16, 17. Therefore, to study the mechanism by which RHOV regulates LUAD cell proliferation, migration, invasion, we explored the relationship between RHOV and JNK pathway in LUAD cells. RHOV overexpression activated phosphorylation of JNK and its downstream target c-Jun, without the changes of expression levels of total JNK and c-Jun in A549 cells (Figure 5A). Pyrazolanthrone, a JNK inhibitor, abrogated RHOV-promoted phosphorylation of JNK and c-Jun. Since EMT is critical for invasion and metastasis of cancer cells 18, we tested the effects of RHOV on EMT in LUAD cells. RHOV overexpression decreased expression of the epithelial marker E-cadherin, and increased that of the mesenchymal marker N-cadherin and the EMT-related transcriptional factors Snail and Slug. However, RHOV overexpression did not change expression of Vimentin, another EMT-related protein, suggesting that RHOV specifically regulates the expression of E-cadherin and N-cadherin. Intriguingly, pyrazolanthrone treatment could also abrogate RHOV-dependent changes of EMT-related proteins (Figure 5A). Cell proliferation and colony formation assays showed that pyrazolanthrone abrogated RHOV promotion of cell proliferation of A549 cells (Figure 5B and 5C). Wound-healing and transwell assays showed that pyrazolanthrone abrogated RHOV promotion of migration and invasion of A549 cells (Figure 5D and 5E). Similar results were also obtained in H1299 cells (Figure S5). Taken together, these results suggested that RHOV promotes LUAD cell proliferation, migration, invasion, and regulates the expression of EMT-related genes, via JNK pathway.

### Knockdown of RHOV suppresses LUAD tumor growth and metastasis in nude mice

**Figure 6:**
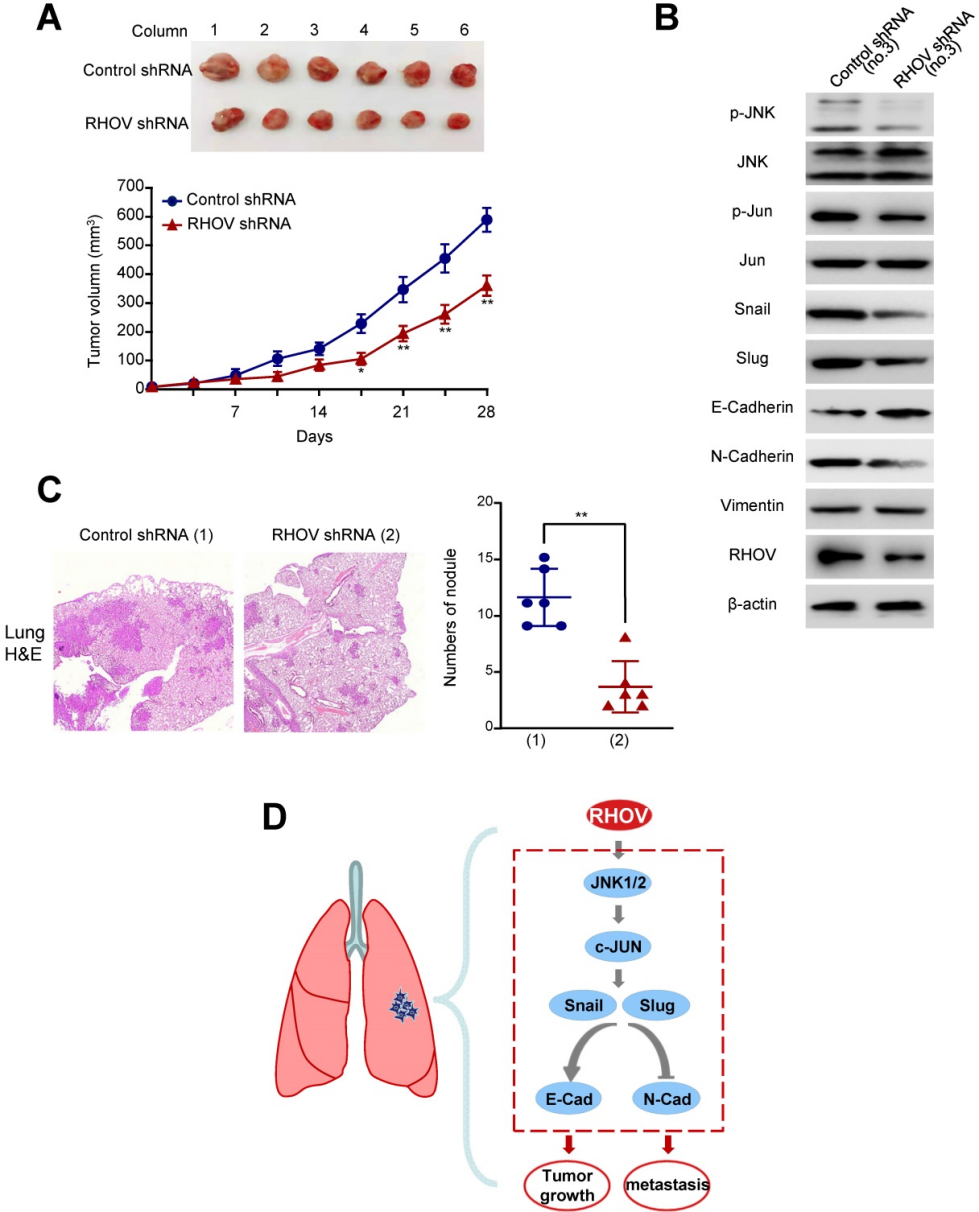
** Knockdown of RHOV suppresses LUAD tumor growth and metastasis in nude mice. (A)** A549 cells stably infected with RHOV shRNA or control shRNA were injected subcutaneously in the right flank of nude mice. At the indicated times, the volume of the tumors was examined with Vernier calipers. **(B)** Immunoblot analysis of representative excised tumor tissues from (A). **(C)** Representative H&E-stained sections of the lung tissues are shown. Data are shown as mean ± SD (n = 6) (*P<0.05, **P<0.01 versus control shRNA).

As RHOV promotes LUAD cell proliferation *in vitro*, we investigated whether RHOV modulates LUAD tumor growth in nude mice. A549 cells stably expressing RHOV shRNA or control shRNA were injected subcutaneously in the right flank of each nude mice. Compared with control shRNA, the tumors with RHOV knockdown grew slowly (Figure 6A). As expected, the A549 tumors in mice inoculated with RHOV shRNA decreased phosphorylation of JNK and c-Jun and expression of N-cadherin, Snail and Slug, and increased expression of E-cadherin (Figure 6B).

Since RHOV promotes LUAD cell migration and invasion *in vitro*, we explored whether RHOV regulates LUAD metastasis. The control and RHOV knockdown A549 cells were suspended in PBS and injected into nude mice via the tail vein. Eight weeks after the injection, H&E staining images of lung metastases demonstrated that RHOV knockdown significantly decreased metastasis of A549 cells (Figure 6C). Taken together, these data suggests that RHOV knockdown inhibits tumor growth and metastasis of LUAD* in vivo*.

## Discussion

In this study, five hub genes, including RHOV, ZIC5, CYP4B1, GPR18 and TCP10L2, have been identified as LUAD metastasis driver genes. Univariate and multivariate Cox regression analysis suggest that RHOV is the most significant gene associated with prognosis of LUAD patients among five genes. RHOV is overexpressed in LUAD patients, and high RHOV expression correlates with large tumor size, advanced clinical stage, lymph node metastasis, and shorter OS. Furthermore, we show that RHOV promotes LUAD cell proliferation, migration, invasion and metastasis both* in vitro* and *in vivo*. The JNK inhibitor pyrazolanthrone diminishes the effect of RHOV on LUAD cell proliferation, migration and invasion, suggesting that RHOV regulates LUAD cell proliferation, migration and invasion via JNK pathway. Thus, our study identifies RHOV as a critical gene responsible for LUAD growth and metastasis, and RHOV may be an ideal target for LUAD therapy.

RHOV is a member of the Rho GTPase family, and plays a vital role in neurodevelopment and embryogenesis 19, 20. RHOV have been identified to activate p21-activated kinases (Paks) 9, 21, which affect a wide range of physiological and pathological processes 22. However, the biological function of RHOV in human cancer is unclear. We show that RHOV controls human LUAD growth and metastasis. JNK is typical serine/threonine kinases and regulates diverse aspects of tumorigenesis and progression, including proliferation, survival and metastasis 23-25. JNK pathway maintains various capabilities of cancer stem cell properties, including self-renewal, drug resistance, and tumorigenesis 26. JNK promotes tumor metastasis via disintegrating adherent junctions 27. The transcription factor c-Jun phosphorylation was identified to be an essential prerequisite for the activation of JNK pathway 17. Phosphorylation of c-Jun can enhance its binding to the promoters of oncogenes, thereby increasing its transcriptional activity 28. EMT, a process involved in tumor metastasis, can also be induced by JNK pathway 29. In this study, we demonstrated that RHOV acts as an oncogene by increasing expression of the EMT-related proteins N-cadherin, Snail and Slug, and decreasing that of the EMT-related protein E-cadherin. Importantly, we revealed for the first time that RHOV promotes growth, EMT and metastasis through JNK phosphorylation in human cancer. Whether RHOV regulates cancer cell growth and metastasis through other signaling pathways remains to be investigated.

Besides RHOV, other four hub genes (ZIC5, CYP4B1, GPR18 and TCP10L2) were screened to be associated with LUAD metastasis in this study. ZIC5 was reported to be highly upregulated in non-small cell lung cancer (NSCLC) tissues and act as an oncogene through regulating the expression of cell cycle-related cdk1/cyclin B1 complex 30. CYP4B1 is a xenobiotics metabolism enzyme, and participates in various biological processes, especially tumorigenesis 31, 32. Compared to normal tissues, CYP4B1 expression level is reduced in the lung tumors 33. GPR18, a cannabinoid-stimulated G protein-coupled receptor, has been implicated in a variety of cancers, involving melanoma 34, breast cancer 35, oral cancer 36, and hepatocellular carcinoma 37. However, the functions of GPR18 have not been yet investigated in lung cancer. TCP10L2 is considered as a pseudogene, and its function remains to be studied.

## Supplementary Material

Supplementary figures and table.
