# Supplementary material for: RHOV promotes lung adenocarcinoma cell growth and metastasis through JNK/c-Jun pathway
Source: Int J Biol Sci. 2021 Jun 22;17(10):2622–32. doi: 10.7150/ijbs.59939 (PMC8315012; doi:10.7150/ijbs.59939)
Supplement: Supplementary file 1 — Supplementary figures and table. [file ijbsv17p2622s1.pdf]

Figure S1

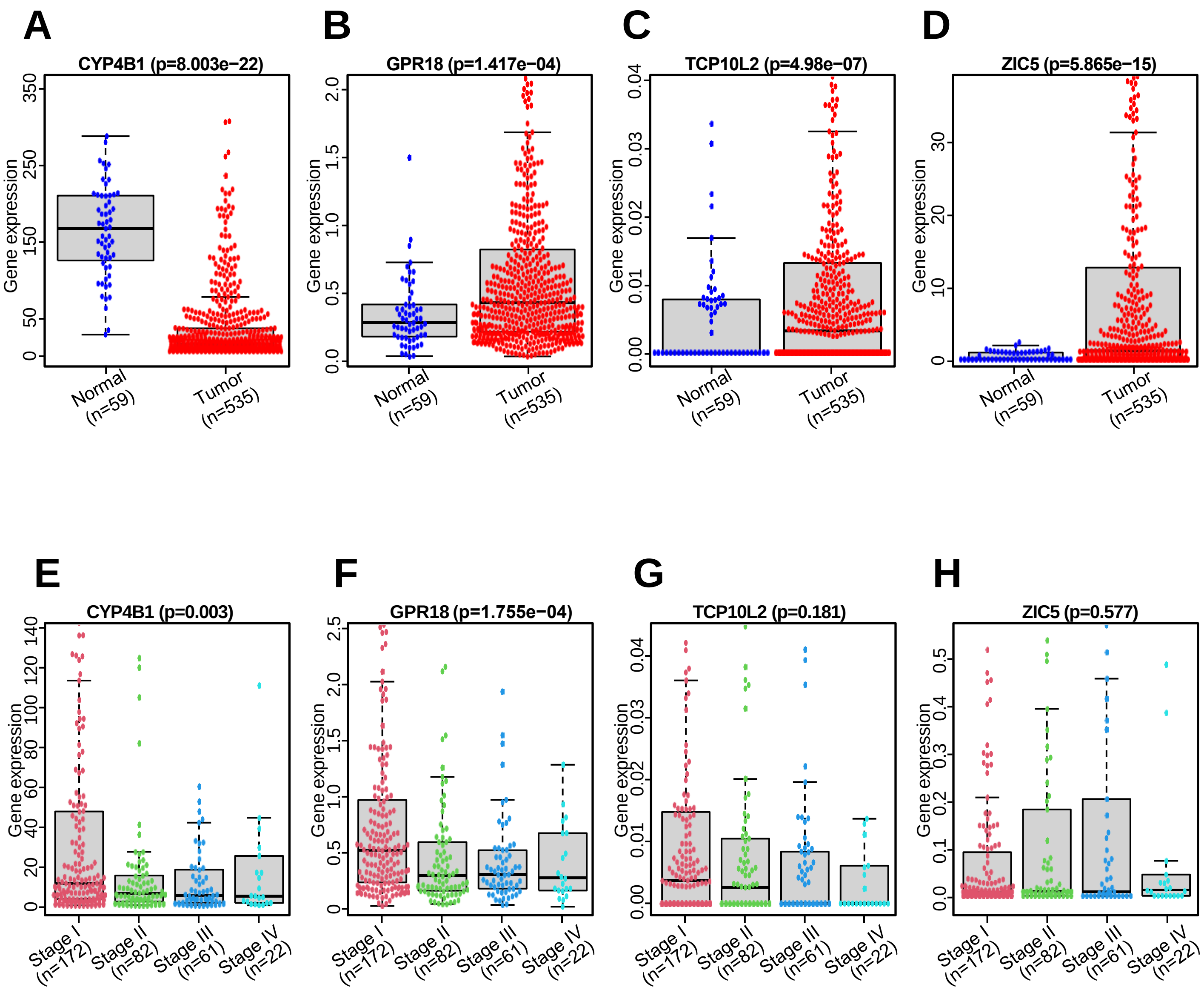

Figure S1. The validation of four hub genes in LUAD patients from TCGA-LUAD dataset.
(A-D) The mRNA expression level of CYP4B1 (A), GPR18 (B), TCP10L2 (C) and ZIC5 (D) in normal tissues and LUAD tissues from TCGA-LUAD dataset. (E-H) The association between the mRNA expression level of CYP4B1 (E), GPR18 (F), TCP10L2 (G) or ZIC5 (H) and clinical stage in TCGA-LUAD dataset.

Figure S2

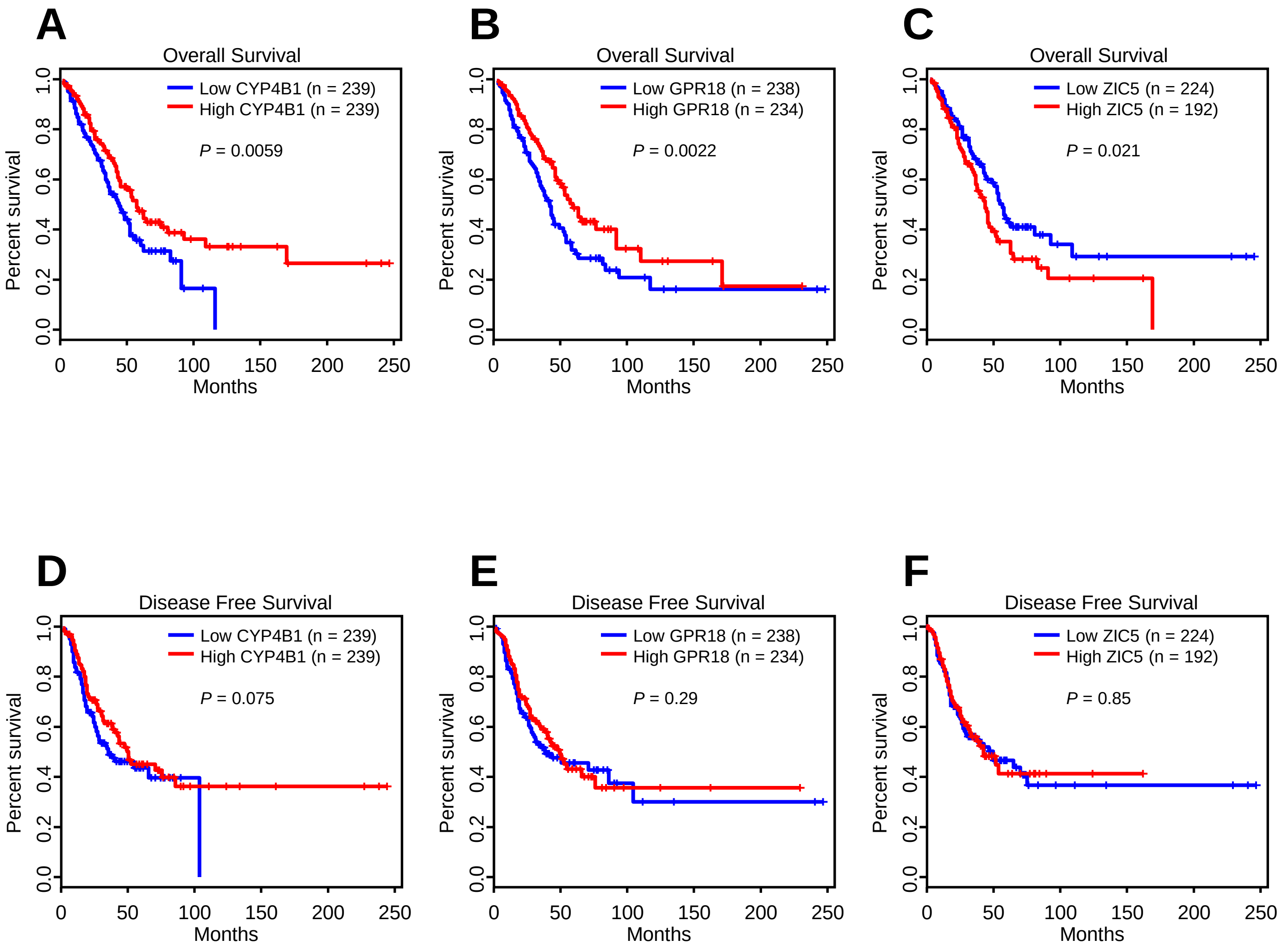

Figure S2. The association between five hub genes and disease-free survival in LUAD patients.
(A-C) The associations between CYP4B1 (A), GPR18 (B) or ZIC5 (C) and overall survival in LUAD patients from TCGA-LUAD dataset. (D-F) The associations between CYP4B1 (D), GPR18 (E) or ZIC5 (F) and disease free survival in LUAD patients from TCGA-LUAD dataset.

Figure S3

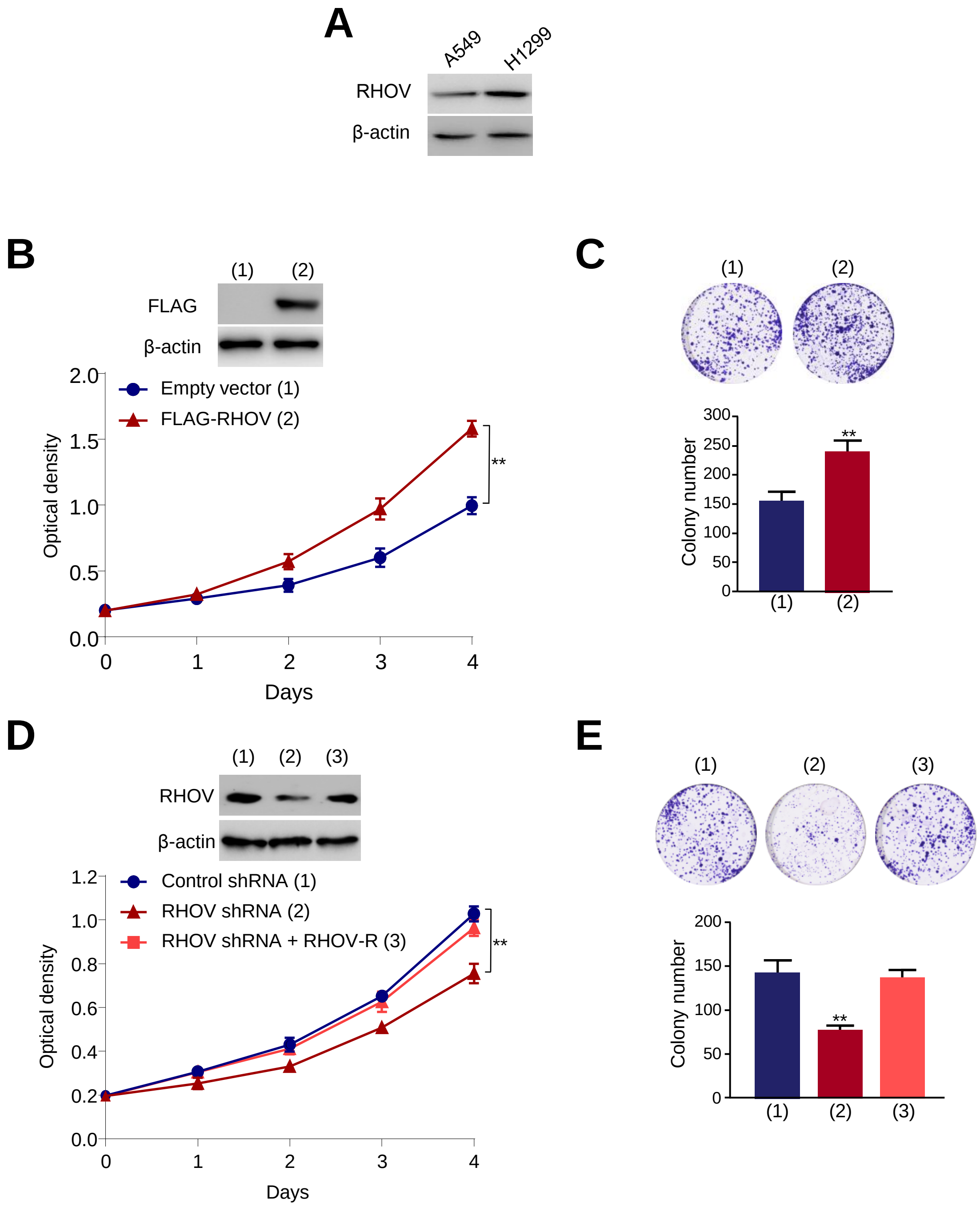

Figure S3. RHOV promotes cell proliferation in H1299 cells.
(A) Total proteins extracted from two LUAD cell lines were analyzed by immunoblotting with anti-RHOV. (B) H1299 cells infected with FLAG-tagged RHOV plasmid or empty vector were grown in regular medium. Cell numbers were detected at the indicated times. Representative immunoblot demonstrates the expression of FLAG-RHOV. (C) Colony formation assays for H1299 cells infected as in (B). (D) H1299 cells were infected with control shRNA, RHOV shRNA or RHOV shRNA plus siRNA-resistant RHOV (RHOV-R), and analyzed as in (B). Representative immunoblot shows RHOV expression. (E) Colony formation assays for H1299 cells infected as in (D). Data shown are mean ± SD of triplicate measurements with similar results (**P* < 0.05, ***P* < 0.01).

Figure S4

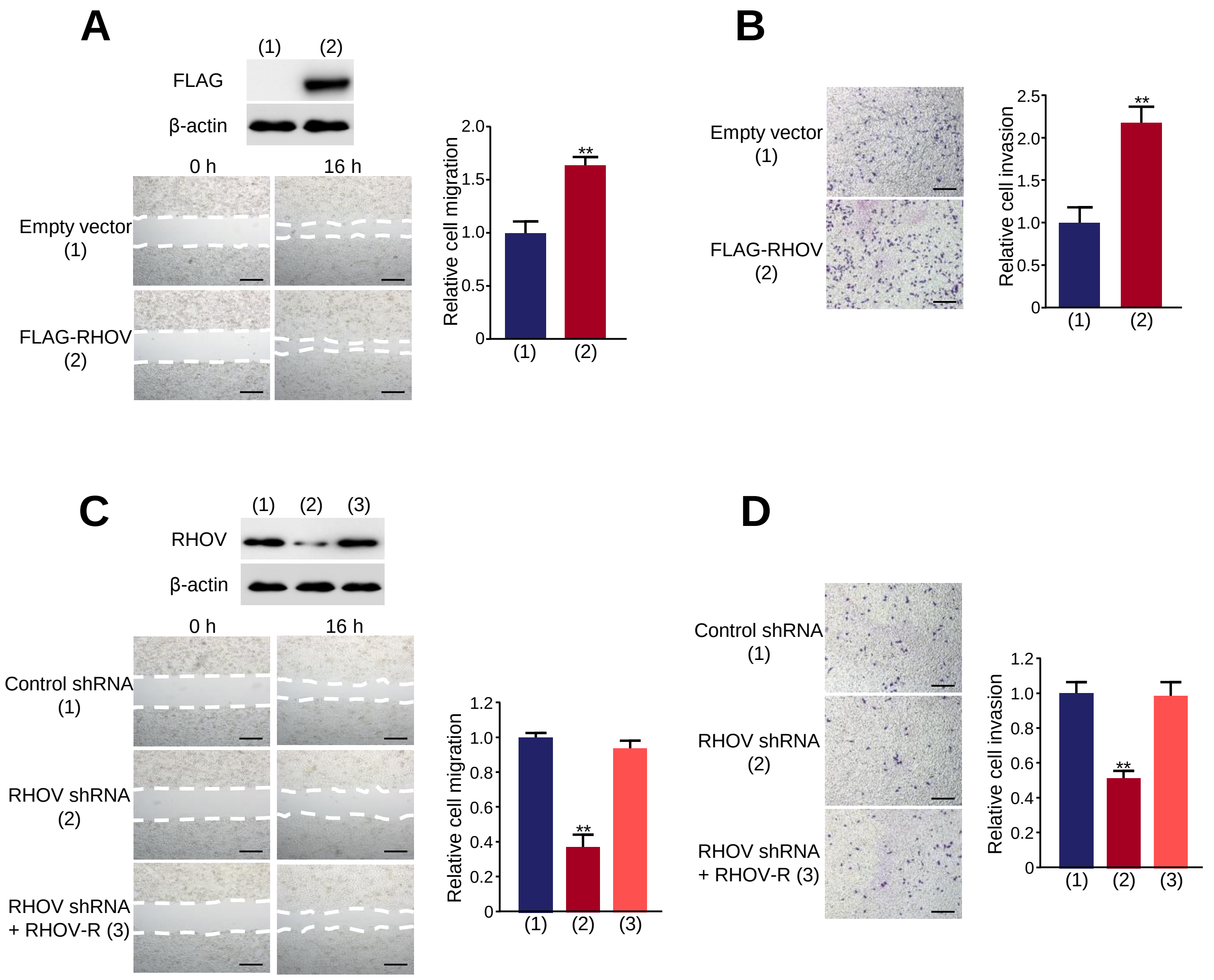

Figure S4. RHOV promotes migration and invasion in H1299 cells.
(A and B) Migration (A) and invasion (B) of H1299 cells overexpressing FLAG-tagged RHOV plasmid or empty vector were evaluated by wound-healing assays and transwell assays, respectively. Scale bar: 100 μm. Representative immunoblot demonstrates the expression of FLAG-RHOV. (C and D) Migration (C) and invasion (D) of H1299 cells infected with control shRNA, RHOV shRNA or RHOV shRNA plus siRNA-resistant RHOV (RHOV-R) were evaluated by wound-healing assays and transwell assays, respectively. Scale bar: 100 μm. Representative immunoblot shows RHOV expression. Data shown are mean ± SD of triplicate measurements with similar results (*$P < 0.05$, **$P < 0.01$).

Figure S5

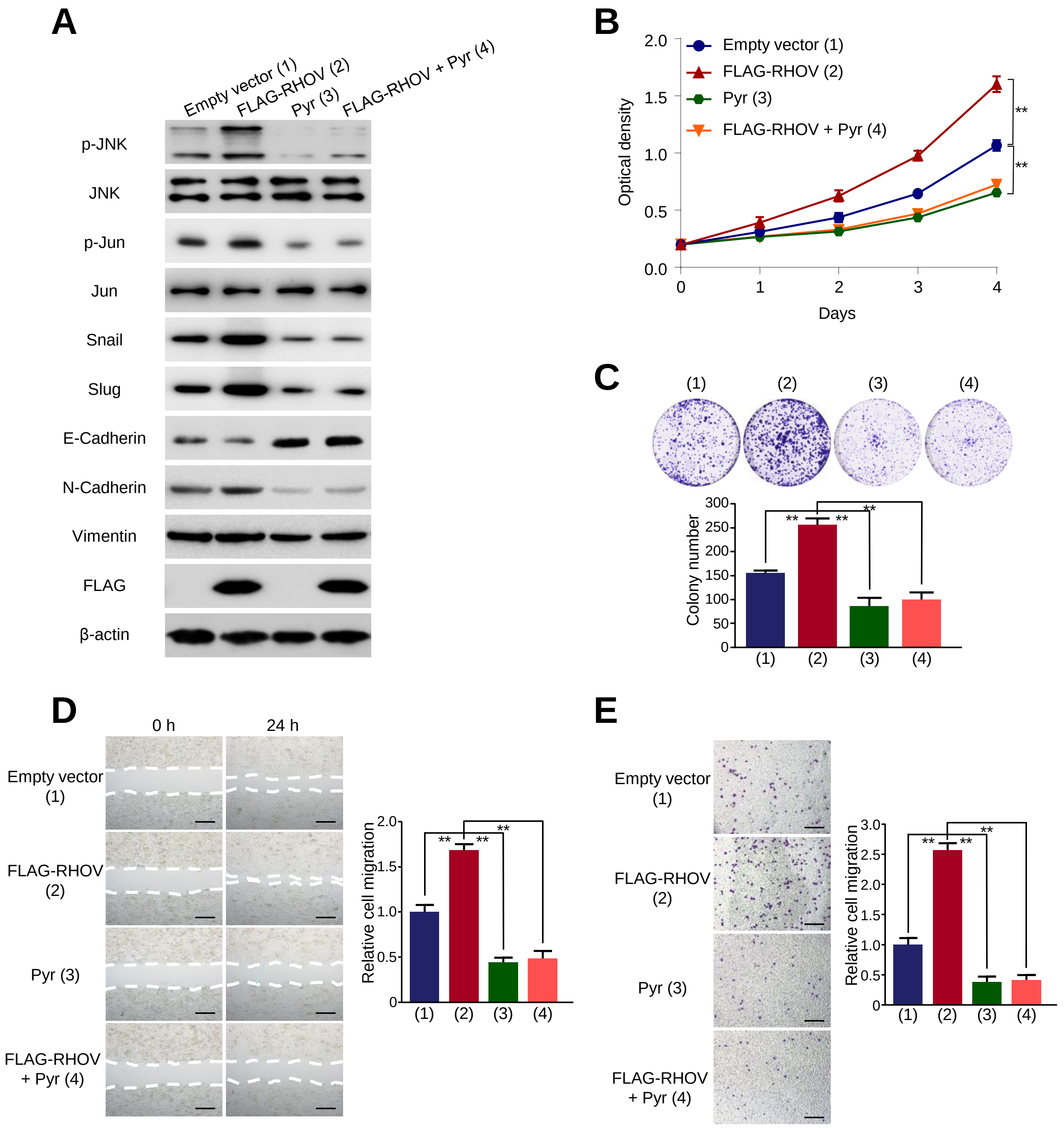

Figure S5. RHOV modulates EMT-related proteins via JNK pathway.
(A) Western blot analysis of H1299 cells infected with FLAG-tagged RHOV plasmid and treated with the JNK inhibitor pyrazolanthrone (10 µM). (B and C) H1299 cells treated as in (A), and cell numbers (B) and cell colonies (C) were detected at the indicated times. (D and E) Migration (D) and invasion (E) of H1299 cells treated as in (A) were evaluated by wound-healing and transwell assays, respectively. Scale bar: 100 µm. Data shown are mean ± SD of triplicate measurements with similar results (**P* < 0.05, ***P* < 0.01).

Table S1. Characteristics of 402 patients from the TCGA-LUAD dataset

Characteristic	non-metastatic predisposition (217 patients / 231 samples)	metastatic predisposition (185 patients / 185 samples)	*P* value
Age (mean ± SD)	65.31±10.28	64.49±9.80	0.4281
Sex (male/female)	101/116	93/90	0.394
Pathologic_M (M1/M0/NA)	0/217/0	25/123/37	<0.01**
Pathologic_N (N3/N2/N1/N0/NA)	0/0/0/217/0	2/74/95/11/3	<0.01**
Pathologic_T (T4/T3/T2/T1/NA)	5/17/114/81/0	13/17/117/36/2	<0.01**
Stage (IV/III/II/I/NA)	5/25/183/4	25/79/79/0/2	<0.01**
Survival status (dead/alive/NA)	59/153/5	100/81/4	<0.01**
